# The aryl hydrocarbon receptor and kynurenine pathway: connecting inflammatory bowel disease and depression via the microbiota gut brain axis

**DOI:** 10.3389/fnins.2026.1851943

**Published:** 2026-10-05

**Authors:** Raymond Bogdon, Kasie Roark, Philip Brandon Busbee

**Affiliations:** Department of Pathology, Microbiology, and Immunology, University of South Carolina Floyd School of Medicine, Columbia, SC, United States

**Keywords:** aryl hydrocarbon receptor, depression, inflammatory bowel disease, kynurenine pathway, microbiota gut-brain axis

## Abstract

Inflammatory bowel disease (IBD) is a disorder characterized by chronic inflammation in the gastrointestinal (GI) tract. This disease has a complex etiology with major contributing factors including genetics, host-microbiome interactions, dysregulation of the immune response, and various environmental triggers. The negative impacts of IBD are not limited to the GI tract however, as IBD patients also develop extraintestinal manifestations involving multiple organs and organ systems. In support of this, IBD patients have increased incidence of mood and behavioral disorders, such as depression, which are often attributed to the concept known as the microbiota gut brain axis (MGBA). This concept refers to the bidirectional communication between the GI tract and central nervous system (CNS) via key components. While it is clear IBD and depression often manifest together, it remains unclear what major mechanisms drive this relationship. Growing evidence suggests that the aryl hydrocarbon receptor (AhR) impacts disease severity in both IBD and behavioral disorders. The purpose of this review is to summarize IBD and depression with emphasis on the potential role AhR plays in both disorders. In addition, evidence of the kynurenine pathway (KP) in IBD and depression will be discussed with particular focus on how this biochemical pathway is linked to both the activation of AhR and its regulation, thus making it an interesting connection requiring further investigation. This review will also summarize key mechanisms of the MGBA implicated in the comorbidity of IBD and depression highlighting the impact, if any, AhR and KP play in these factors. Lastly, the review will discuss limitations of this current topic and any potential future directions to better understand the roles of AhR and KP in comorbid IBD and depression.

## 1 Introduction

Inflammatory bowel disease (IBD) is characterized by chronic inflammation of the gastrointestinal (GI) tract attributed to a combination of interplaying factors such as genetics, environmental triggers, luminal microbiota, and dysregulation in the immune response (Abegunde et al., 2016). IBD patients often have increased incidence of comorbid behavioral and mood disorders (Yuan et al., 2021; Marrie et al., 2021; Kochar et al., 2018). While not fully understood, the comorbidity between IBD and depression is linked to the bidirectional communication between the GI tract and central nervous system (CNS) known as the microbiota gut brain axis (MGBA). However, while there is clear evidence that IBD can promote depression (Bisgaard et al., 2023; Ma et al., 2023), and vice versa (Kochar et al., 2018; Gao et al., 2021; Ruan et al., 2023), factors which contribute to this relationship are not fully understood, nor has a therapeutic target been identified which might address both conditions simultaneously.

A report was published in 2016 showing IBD patients had decreased expression and activation of the aryl hydrocarbon receptor (AhR), a cytosolic-bound receptor and transcription factor (Lamas et al., 2016). In addition, AhR knockout (KO) mice develop more severe symptoms in preclinical IBD models (Furumatsu et al., 2011), highlighting the relevance of AhR to this disease. Interestingly, AhR is linked to tryptophan (Trp) metabolism, a process primarily performed in mammalian host cells through the kynurenine pathway (KP) (Shen et al., 2023). Like AhR, Trp metabolism and the KP have been implicated in both IBD (Michaudel et al., 2023) and depression (Lin et al., 2023; Li et al., 2023), providing strong correlative evidence of the importance of AhR and KP in both conditions. Despite the exciting potential for exploring AhR and the KP in the comorbidity of IBD and depression, much of the evidence linking these mechanisms to these diseases is limited to preclinical animal models, and even more importantly, often these mechanisms are investigated separately in either IBD or depression studies, not in tandem. The purpose of this review will be to summarize the growing evidence linking IBD and depression to Ahr and KP signaling. After briefly summarizing key factors of IBD and depression, this review will discuss AhR, the KP, the connections between these processes, and highlighting their potential roles in regulating key mechanisms linked to comorbid IBD and depression, such as microbial dysbiosis/metabolomic destabilization, GI-related immune dysregulation/barrier disruption, systemic inflammation, and blood brain barrier (BBB) disruption/neuroinflammation. By doing so, the review will attempt to “connect the dots” regarding what is currently known about AhR and KP involvement in the MGBA as it relates to comorbid IBD and depression. Lastly, we will discuss any current limitations for exploring the role of AhR/KP in the comorbidity of IBD and depression, with considerations for potential future directions regarding this topic.

## 2 Inflammatory bowel disease (IBD)

### 2.1 Background

IBD is a group of chronic GI disorders with unclear etiology, resulting in socially isolating symptoms and a reduction in patient quality of life (QoL) (Knowles et al., 2018). The incidence of IBD has consistently climbed, with prevalence in the industrialized world estimated at 0.5% of the general population (Molodecky et al., 2012), and is increasingly becoming more documented in developing nations making this a significant global health burden (Kaplan, 2015). IBD is comprised of two main types: Crohn's disease (CD) and ulcerative colitis (UC). CD can manifest in any portion of the GI tract in a discontinuous manner, whereas UC is confined to the colon usually in small patches (Flynn and Eisenstein, 2019). Symptomatically both UC and CD often present with diarrhea and abdominal pain, but bloody stool and rectal bleeding are more common in UC (Yu and Rodriguez, 2017). Ulcerations are also common manifestations in both diseases, although ulcers in UC are restricted to the mucosa whereas in CD they often penetrate deeper into the intestinal tissue layers (Podolsky, 2002). Diagnostic criteria for IBD can be assessed either with stool or blood analyses (Cao et al., 2021), but final confirmation of UC or CD is usually determined via colonoscopy or endoscopy respectively, although ultrasound technology has recently been considered (Ilvemark et al., 2026).

### 2.2 Standard treatment and unmet challenges

While there is currently no cure for IBD, there are treatments which can alleviate symptoms and improve the patient's QoL. Biologic agents using monoclonal antibody (mAB) targeting of tumor necrosis factor (TNF)-α, such as infliximab, have been a promising therapeutic strategy for IBD, but at least one third of IBD patients are primary non-responders to anti-TNF-α therapy and effectiveness wanes over time (Na and Moon, 2019). Janus kinase (JAK)-1 inhibitors like upadacitinib are prescribed to reduce the downstream inflammatory influence of IL-6 activation via the JAK-signal transducer and activator of transcription 3 (STAT3) pathway, but adverse events like secondary infections or deep vein thrombosis have been reported (Sandborn et al., 2020). Anti-IL-12/23 mABs like ustekinumab control intestinal inflammation by blocking the ability to bind to IL-12 receptors on immune cells (Rutgeerts et al., 2018). Vedolizumab, an anti-integrin mAB, inhibits lymphocyte migration into tissue by blocking α4β7 integrin binding to the mucosal addressin cell adhesion molecule-1, but potentially is more efficacious in patients who have not received previous anti-TNF-α therapy (Feagan et al., 2019). Immunomodulators, like methotrexate, are sometimes employed along with TNF-α inhibitors, but carry with them the possibility of severe side-effects (Vasudevan et al., 2020). Steroids, such as immunosuppressive corticosteroids, can raise the risk of secondary infections, and patients often develop resistance after prolonged use (Alam et al., 2023). Aminosalicylates like mesalamine act as a reactive oxygen species (ROS) scavenger, modulate cytokine expression, and increase Treg accumulation in the colon (Oh-Oka et al., 2017). Lastly, antibiotics (Abx) have been prescribed for mild disease presentation, but often lead to moderate side effects and have been shown to inconsistently induce remission (Su et al., 2015; Dye et al., 2025). Even with diverse treatment options, nearly 50%−80% CD patients and 10%−30% UC patients still must undergo surgical intervention (Khoudari et al., 2022) which highlights the need for ongoing research and identification of alternative treatment options.

### 2.3 IBD contributing factors and association with depression

Historically, researchers have highlighted several potential triggers for the onset of IBD such as microbial dysbiosis, immune modulation, autonomic dysfunction, and psychological distress (Tavakoli et al., 2021). A recent meta-analysis of risk factors for IBD identified several environmental or host-lifestyle factors that increased one's risk such as: smoking, urban living, appendectomy, Abx exposure, oral contraceptives, vitamin D deficiency, and non-*Helicobacter pylori*-like enterohepatic *Helicobacter* species infection (Piovani et al., 2019). In addition, over 200 genetic loci have been implicated in IBD. Genes with the strongest associations in humans were involved in the immune response to microbes, such as nucleotide-binding oligomerization domain-containing protein (NOD)2, interleukin (IL)-23R, and autophagy related (ATG)16L1 (Jostins et al., 2012). Microbial dysbiosis, characterized by a reduction in gut microbial diversity due to a shift in the balance between commensal and pathogenic microbiota, has been identified as a crucial factor in IBD development (Ott et al., 2004; Lopetuso et al., 2023). Such alterations in the gut microflora can lead to degradation of the mucus barrier creating a permissible environment for bacterial invasion and increased immune cell activation (Shan et al., 2022). Additionally, there exists evidence of sex-specific differences in the pathogenesis and clinical outcomes of IBD as recently reviewed by Salem et al. (2024).

In addition to contributing factors to IBD, IBD patients often experience extraintestinal manifestations (EIMs), such as increased susceptibility to development of depression. For example, a systematic study found that 20% of IBD patients had symptoms of depression and active flare ups increased this prevalence to 40% (Neuendorf et al., 2016). Indeed, this was corroborated in a second study which found that active episodes of gut inflammation were associated with higher psychiatric metrics in IBD patients (Abautret-Daly et al., 2017). Comparatively, a recent meta-analysis found that 24% of IBD patients had depression, but active symptoms increased this to 41.3% (Barberio et al., 2021). Interestingly, there is also evidence which suggests that patients with a history of depression were more likely to develop IBD, but antidepressant treatment was found to reduce the likelihood of this occurring (Frolkis et al., 2019). To better understand the links between IBD and depression, it is important to note key features of this mood disorder and find the common factors associated with both.

## 3 Depression

### 3.1 Background

Depression is thought to be brought on by combination of psychosocial or biological stressors (Menard et al., 2016) and presents clinically as a variety of disorders such as: major depressive disorder (MDD) (Kupfer et al., 2012), psychotic depression (Dubovsky et al., 2021), persistent depressive disorder (Schramm et al., 2020), postpartum depression (Agrawal et al., 2022), or bipolar depression (BPD) (Tondo et al., 2017). Approximately 280 million people worldwide are affected by depression with 21 million Americans over the age of 18 experiencing at least one major depressive episode (McCarron et al., 2021). A recent meta-analysis observed a stratification of depression prevalence by age with 8% of adolescents (Shorey et al., 2022), 2%−21% of adults (Gutierrez-Rojas et al., 2020), and 13.3%−35.1% of the elderly (Cai et al., 2023) suffering from clinically relevant depressive symptoms. It is important to note that depressive symptoms are different from normal mood fluctuations, often being more intense and recurrent which result in greatly diminished QoL. Patients typically experience anhedonia, fatigue, weight loss, insomnia, feelings of worthlessness or guilt, suicidal ideation, and depressed mood. Symptoms are not uniform between patients either, making depression difficult to identify. Current diagnostic screenings for depression rely heavily on patient reporting as there are no useful biological diagnostic markers or biological screening tests currently available (McCarron et al., 2021), although research has been conducted to assess metabolic alteration signatures in the patient population (Jansen et al., 2024).

### 3.2 Standard treatments and unmet challenges

Treatment options for clinical depression often involve pharmaceutical interventions paired with focused psychotherapy with a certified clinician such as cognitive behavioral therapy (CBT) or interpersonal psychotherapy (IPT) (Yasinski et al., 2026). Regarding pharmaceuticals, Cipriani et al. (2018) performed a comparative analysis of over 522 trials and 21 standard anti-depressant drugs to treat acute depression in adults, finding that all were more efficacious than placebos. However, approximately 30% of patients with categorized clinical depression do not achieve remission after treatment with two or more first-line antidepressants and are considered to have treatment resistant depression (TRD) (Pompili et al., 2026). Since the 1960s, abnormalities in neurotransmitters, mainly the Trp derivative serotonin, were thought to be a major contributing factor to depression. More recently, the effectiveness of selective serotonin reuptake inhibitor (SSRI) antidepressants was found to have limited overall effect according to a metanalysis by Moncrieff et al. (2023).

### 3.3 Depression contributing factors and common features with IBD

The risk factors for clinical depression are varied and include personal or family history, biological sex (female), alcohol/drug dependency, childhood trauma, recent childbirth, low socioeconomic status, and chronic medical illness (McCarron et al., 2021). Over the years, many hypotheses have aimed to explain the pathogenesis of this condition resulting in a range of potential contributing factors such as genetic and receptor abnormalities (Wang et al., 2021), monoamine depletion (Heninger et al., 1996), neurotrophic (Tartt et al., 2022), cholinergic (Mineur and Picciotto, 2010), hypothalamic-pituitary-adrenal (HPA) axis dysregulation (Cheiran Pereira et al., 2022), MGBA dysfunction (Kim et al., 2023), and inflammation (Suneson et al., 2021). Taken as a whole, it is far more likely that a combination of the above factors cooperatively induces this mood disorder. However, it is important to note that key contributing factors mentioned for depression are also associated with IBD, particularly inflammation, MGBA dysfunction, and Trp metabolism imbalance. As is highlighted in the proceeding sections, all of these processes can be linked to AhR.

## 4 Aryl hydrocarbon receptor (AhR)

### 4.1 Background

AhR is a ligand-activated receptor and transcription factor which has been highly conserved throughout evolution (Hahn et al., 1997). AhR is a member of the basic helix-loop-helix/Per-ARNT-SIM family of transcription factors and, from N- to C-terminus, consists of the following key domains: a basic helix-loop-helix domain responsible for DNA binding, two Per-ARNT-SIM (PAS) domains arranged in tandem (PAS-A and PAS-B) to facilitate dimerization, and a transcription activation domain (Fukunaga et al., 1995; Dai et al., 2022). AhR is stabilized in the cytoplasm by the molecular chaperone complex HSP90-p23-XAP2 (Uemura et al., 2020). Ligand binding leads to nuclear translocation as the AhR-chaperone complex dissociates and AhR heterodimerizes with the AHR nuclear translocator (ARNT). The AhR-ARNT complex then binds to AHR response elements known as dioxin response element (DRE) or xenobiotic response element (XRE) in the canonical pathway (Haidar et al., 2021). Once bound to the response element, AhR-ARNT complex functions as a transcriptional complex that regulates and modifies transcriptional activity and chromatin structure (Wajda et al., 2020). An illustrated summary of the key events in activation of canonical and non-canonical AhR pathways (Wright et al., 2017) are summarized in Figure 1.

**Figure 1:**
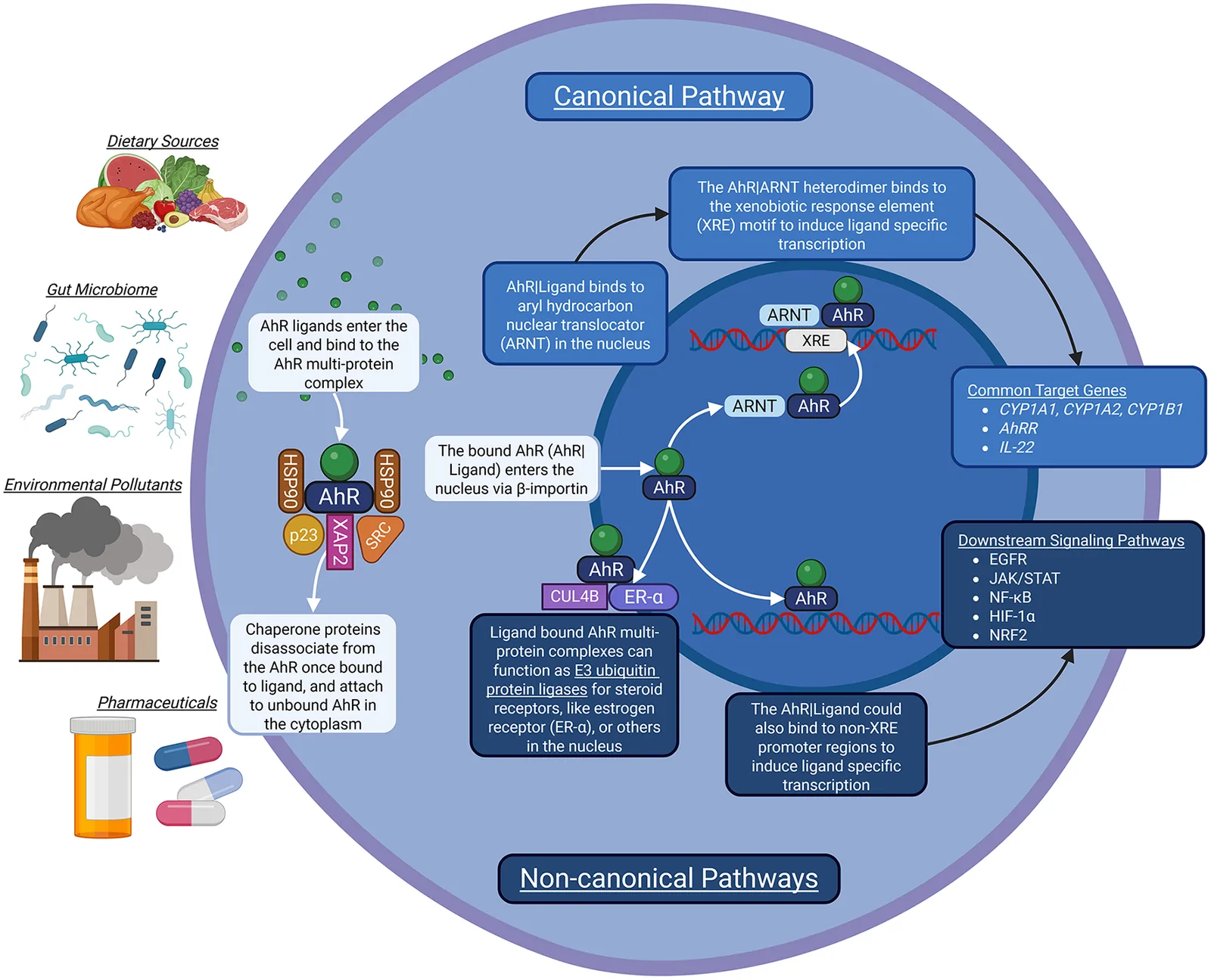
Activation of the aryl hydrocarbon receptor pathway. The aryl hydrocarbon receptor (AhR) is a cytosolic transcription factor bound within a multi-protein complex comprised of: 2 heat shock protein 90 (HSP90), AhR-interacting protein (XAP2), co-chaperone p23 (p23), and protein tyrosine kinase c-Src (SRC). Once bound to a ligand the multi-protein complex disassociates from the bound AhR, and can then conjugate with unbound AhR in the cytosol. The bound AhR then undergoes nuclear transport with assistance from β-importin. At this point, the AhR pathway can broadly be divided between canonical and non-canonical. In the canonical pathway the AhR nuclear translocator (ARNT) combines with AhR, and at this point the dimer can bind to xenobiotic-responsive elements (XRE) to modulate genetic expression. In the non-canonical pathway bound AhR are capable of binding to non-XRE promoter regions without assistance from ARNT to induce ligand specific transcription and affect several downstream signaling pathways. In addition, bound AhR can function as E3 ubiquitin protein ligases providing additional regulation for unbound nuclear receptors. Created with Biorender.com.

### 4.2 Ligands of AhR

The PAS-B domain of the AhR is responsible for ligand binding and is structurally flexible to allow a variety of ligands with diverse chemical structures, origins, and binding affinities to bind (Pongratz et al., 1998). Many natural ligands for AhR are derived from host-consumed diets such as resveratrol (polyphenol in skin of red grapes), curcumin (compound in turmeric), indole-3-carbinol (I3C) (indole in cruciferous vegetables), and quercetin (flavonoid in many foods) (Lin C. H. et al., 2022; Guyot et al., 2013; Denison and Nagy, 2003; Bock, 2020). Products formed through the microbial-mediated indole metabolism of Trp also act as AhR ligands such as formylindolo [3,2-b] carbazole (FICZ), indigo, and indirubin (Rannug and Rannug, 2018; Kim et al., 2024). Some synthetic exogenous AhR ligands derived from environmental pollutants have shown high binding affinity and substantial levels of toxicity. These include polycyclic aromatic hydrocarbons such as benzo(a)pyrene, halogenated aromatic hydrocarbons like 2, 3, 7, 8-tetrachlorodibenzo-pdioxin (TCDD), polychlorinated dibenzo-p-dioxins, and biphenyls (Hammond et al., 2022). KP metabolites like kynurenine (KYN) or kynurenic acid (KYNA) can be microbial or host-derived and will be expanded upon later in this review (Shadboorestan et al., 2023). A select list of common AhR ligands and their sources has been summarized in Table 1.

**Table 1:** Source and examples of AhR ligands.

Origin	Source	Examples
Natural	Dietary	Resveratrol Curcumin Indole-3-Carbinol (I3C) Quercetin Kynurenine (KYN) Kynurenic acid (KYNA) Cinnabarinic acid (CA) Xanthurenic acid (XA) 6,2,4′-trimethoxyflavone (TMF) 6-Formylindolo (3,2-b)carbazole (FICZ) Diindolymethane (DIM)
	Microbiota	Butyrate Indole-3-acetic acid (IAA) Indole-3-propionic acid (IPA) 1,4-Dihydroxy-2-naphthoic acid (1,4-DHNA) 5,6,7-Trihydroxyflavone (Baicalein) Indole Indolo[3,2-b]carbazole (ICZ) Tryptamine (TRYP)
Synthetic	Environmental pollutant	2,3,7,8-Tetrachlorodibenzofuran (TCDF) 2,3,7,8-tetrachlorodibenzo-pdioxin (TCDD) 1,2-Benzophenanthrene (Chrysene) (CHR) Dibenz[a,h]anthracene [D(a,h)A] Indeno[1,2,3-cd]pyrene 7,12-dimethylbenz[a]anthracene (DMBA) Coal tar Diesel exhaust particles (DEP)
	Pharmaceutical	(Z)-2-(3,4-dichlorophenyl)-3-(1H-pyrrol-2-yl)acrylonitrile (ANI-7) Avitriptan Carbidopa Doxorubucin Guanabenz (Wytensin) Ketoconazole (KCZ) Omeprazole 6-amino-2-phenylchromen-4-one (Aminoflavone) (Z)-2-(2-aminophenyl)-1H-benzo[de]isoquinoline-1,3(2H)dione (NAP-6)

### 4.3 Distribution and role of AhR

Studies show AhR is expressed in a variety of tissues and cell types throughout the body. For example, AhR is expressed in varying degrees in the liver, gut, skin, lungs, and brain (Bernshausen et al., 2006; Petersen et al., 2000). Additionally, AhR is expressed in specific regions of enteric neurons in the colon and small intestine (Sun et al., 2025). On a cellular level many innate and adaptive immune cells display high expression of AhR including macrophages (Malany et al., 2024), dendritic cells (DC), innate lymphoid cells (ILCs), and certain T helper (Th) subsets (i.e., T-regulatory (Treg), Th17, Th22) (Helm and Zhou, 2023; Cui et al., 2022). Beyond immune cells, and pertinent to the current review, AhR is also expressed in epithelial cells (ECs), neurons, microglia, and glial cells such as astrocytes (Sheu et al., 2023; Filbrandt et al., 2004; Imran et al., 2022; Rothhammer et al., 2021).

AhR was first discovered when researchers identified its modulatory role in response to TCDD, a highly immuno-toxic and -suppressive contaminant of the chemical herbicide Agent Orange in 1976 (Poland et al., 1976). Since then, research has further supported AhR as an important regulator of host-environment interactions (Al-Ghezi et al., 2019; Metidji et al., 2018). Recent findings show AhR can influence both the innate and adaptive immune responses in addition to inflammatory processes (Sun et al., 2022; Huang et al., 2022; Hezaveh et al., 2022; Kenison et al., 2021). AhR also contributes to the regulation of many bodily functions including immune cell fate determination, detoxification, malignant transformation, oxidative stress, hematopoietic stem cell differentiation, and several metabolic processes (Healey et al., 2024; Chen et al., 2022; Puyskens et al., 2020). AhR has been shown to intrinsically promote resident memory CD8+ T-cell differentiation and function, balance gut ILC populations, and regulate CD4+ T-cell and DC differentiation, proliferation, and function (Dean et al., 2023; McAleer et al., 2018; Li et al., 2018). AhR also shares a close relationship with gut microbiota as the breakdown of compounds, such as indoles, provide several AhR ligands which can influence the immune response (Sun et al., 2022), and impaired AhR ligand production by gut microbiota has been shown to be a key component in metabolic syndrome (Natividad et al., 2018). Several studies have identified that AhR dysregulation plays a role in disease promotion, including IBD and depression (Yu et al., 2018; Luo et al., 2023; Debler et al., 2023), which is the focus of the current review.

### 4.4 The role of AhR in IBD and depression

Several AhR ligands are known to be effective at reducing disease severity in preclinical animal models of IBD. Table 2 highlights some of these ligands which were also found to impact animal models of depressive-like behavior, discussed the subsequent paragraph. Such ligands known to impact preclinical models of IBD include TCDD (Singh et al., 2011), FICZ (Yu et al., 2018), I3C (Busbee et al., 2020; Riemschneider et al., 2021), 3,3′-diindolymethane (DIM) (Jeon et al., 2016), resveratrol (Qin et al., 2025), indole-3-acetic acid (IAA) (Li et al., 2024), indole-3-propionic acid (IPA) (Flannigan et al., 2023), indole-3-lactic acid (ILA) (Yu et al., 2023), 1,4-DHNA (Fukumoto et al., 2014), and crocetin (Feng et al., 2022). One study found that I3C or quercetin supplementation was able to ameliorate chronic dextran sodium sulfate (DSS)-induced colitis symptoms in wildtype, but not AhR KO animals, highlighting the necessity of the AhR in attenuation of colitis (Riemschneider et al., 2021). Pernomian et al. (2020) wrote an extensive review highlighting several studies showing AhR dysregulation impacts IBD, and other studies show AhR KO mice develop significantly worse colitis (Furumatsu et al., 2011). In addition, human clinical data has shown that UC and CD patients have decreased expression, signaling, and activation of AhR in the GI tract (Monteleone et al., 2011; Zhao et al., 2016; Lamas et al., 2016). These findings collectively implicate AhR in disease regulation and highlight its potential role as a promising therapeutic target for IBD.

**Table 2:** Use of AhR ligands in preclincial IBD models.

Ligand	Dosage	Model	Impact on disease severity and highlighted mechanisms	References
TCDD	25 μg/kg	DSS-induced colitis	Ameliorate; increases anti-inflammatory Tregs and decreases inflammatory Th17 cells	Singh et al., 2011
FICZ	1 μg	DSS-induced colitis	Ameliorate; enhanced intestinal barrier via tight junction protein expression	Yu et al., 2018
I3C and quercetin	20 mg/kg (I3C), 50 mg/kg (quercetin)	DSS-induced colitis (chronic)	Ameliorate; prevented loss of tight junction proteins (barrier integrity) and reduced gut inflammation (decreased neutrophils, macrophages, and Th17, while increasing Tregs)	Riemschneider et al., 2021
I3C	40 mg/kg	DSS and TNBS-induced colitis	Ameliorate; prevented microbial dysbiosis through IL-22, enhanced Treg response and Muc2 production	Busbee et al., 2020
DIM	20 mg/kg	DSS-induced colitis	Ameliorate; suppressed neutrophil infiltration/proinflammatory cytokines	Jeon et al., 2016
Resveratrol	100 mg/kg	DSS-induced colitis	Ameliorate; enhanced barrier function (tight junction protein expression), suppressed pro-inflammatory cytokines, promoted anti-inflammatory M2 macrophages, and enriched beneficial intestinal bacteria	Qin et al., 2025
IAA	Not specified	DSS-induced colitis	Ameliorate; increased *Bifidobacterium pseudolongum* which promoted anti-inflammatory Tregs	Li et al., 2024
ILA	40 mg/kg	DSS-induced colitis	Ameliorate; decrease epithelial CCL2/7 which reduced inflammatory macrophage infiltration	Yu et al., 2023
IPA	250 mg/L	DSS-induced colitis	Ameliorate; enhancing pregnane X receptor (PXR) signaling which is linked to suppression of intestinal inflammation	Flannigan et al., 2023
1,4-DHNA	2, 20 mg/kg	DSS-induced colitis	Ameliorate; induced intestinal-associated anti-microbial peptides (RegIIIβ/γ) to prevent microbial dysbiosis	Fukumoto et al., 2014
Crocetin	10, 40 mg/kg	DSS-induced colitis	Aggravate; enhanced microbial dysbiosis and reduced primary and secondary bile acids	Feng et al., 2022

While the role of AhR in depression is not as extensive as with IBD, as mentioned in the preceding paragraph, certain AhR ligands were shown to impact depressive-like behavior in a variety of animal models, such TCDD (Debler et al., 2024), FICZ (Wang et al., 2024), I3C (Pan et al., 2022), DIM (Madison et al., 2022b), resveratrol (Ali et al., 2015), IAA/IPA (Wang et al., 2025), ILA (Qian et al., 2024), 1,4 DHNA (Debler et al., 2023), and crocetin (Lin S. et al., 2022) (Table 3). To this point, activation of AhR using β-naphthoflavone (BNF) was shown to rescue depressive-like behavior while AhR KO was shown to induce depressive-like behavior in preclinical murine models (Vicentini et al., 2021). It is also important to note that excessive activation of the AhR could lead to deleterious effects such as exacerbation of the inflammatory reflex or behavioral changes as shown in a spared nerve injury model by Luo et al. In this study, animals were treated with the soluble epoxide hydrolase inhibitor 1-trifluoromethoxyphenyl-3-(1-propionylpiperidin-4-yl) urea (TPPU), and it was determined that the anti-depressant effects were partly AhR-dependent as they were mitigated following administration of AhR agonist FICZ. However, use of an AhR antagonist resulted in similar anti-depressant effects as TPPU treatment (Luo et al., 2023). This was also seen in a preclincal model of chronic kidney disease where mice were administered the potent AhR agonist indoxyl sulfate (IS). Peripheral IS administration resulted in neuroinflammation, reduced neuronal survival, and altered behaviors. Yet, central administration of IS, compared to peripheral, resulted in reduced alterations of neuroinflammation and behaviors which were abrogated with AhR antagonism (Sun et al., 2021). As AhR is more pronounced in the liver and kidneys compared to that of the brain (Juricek and Coumoul, 2018), it is possible that peripherally derived AhR-signaling has a greater influence on behavior than direct central-AhR stimulation. Biological sex could also be a contributing variable, yet there is a current gap of knowledge on this subject. One study identified differing outcomes following administration of 1,4-DHNA, where females experienced anti-depressant effects, but males did not. While remaining mechanistically unclear, 1,4-DHNA did mitigate some stress reactivity in the male cohort, and the researchers hypothesized that this could be a ligand-specific interaction to co-modulatory effects between AhR and ERs in the hippocampus (Madison et al., 2022a).

**Table 3:** Use of AhR in preclinical depressive-like behavior models.

Ligand	Dosage	Model	Impact on disease severity and highlighted mechanisms	References
TCDD	0.1 μg/kg	High-fat diet (HFD)	Aggravate; no mechanisms highlighted	Debler et al., 2024
FICZ	50 mg/kg	Lipopolysaccharide (LPS)-induced depression	Ameliorate; decreased hippocampal and serum pro-inflammatory cytokines (e.g., IL-6, IL-1β) and downregulated NLRP3 in hippocampus	Wang et al., 2024
I3C	10, 30, and 60 mg/kg	Chronic-stressed induced depression	Ameliorate; reduced stress-induced pro-inflammatory cytokines (IL-6, IL-1β, TNF-α) in hippocampus and prefrontal cortex, suppresses oxido-sative stress in the brain	Pan et al., 2022
DIM and 1,4-DHNA	20 mg/kg	Unpredictable chronic mild stress (UCMS)	Ameliorate; no mechanisms highlighted	Madison et al., 2022b
Resveratrol	80 mg/kg	Repeated corticosterone-induced depression	Ameliorate; reduced serum cortisol and increased hippocampal BDNF	Ali et al., 2015
Indole solution (IAA, IPA, indole)	300 μM (with 0.04 mg/kg FICZ)	Fecal microbiota transplantation (FMT) from methamphetamine (Meth) withdrawal	Ameliorate; prevents microglial dystrophy and enhances neurogenesis in hippocampus	Wang et al., 2025
ILA	35 mg/kg	Chronic unpredictable mild stress (CUMS)	Ameliorate; alleviates neuroinflammation (e.g., reduced pro-inflammatory hippocampal cytokines) and reduces serum corticosterone	Qian et al., 2024
1,4-DHNA	20 mg/kg	HDF	Ameliorate; no mechanisms highlighted	Debler et al., 2023
Crocetin	40, 80 mg/kg	CUMS	Ameliorate; decreased colon and serum pro-inflammatory cytokines and enhanced intestinal barrier function (tight junction proteins); reduced hippocampal IDO1 and serotonin transporter (SERT) expression	Lin S. et al., 2022

While interesting, most of the studies investigating the potential role of AhR ligands in IBD or depression are preclinical, and more importantly, not studied in the context of comorbid IBD and depression. Research has shown that the KP and KP-associated metabolites are not only involved in the production of ligands that activate AhR (e.g., KYN/KYNA), but AhR can also regulate key components in the KP (Shadboorestan et al., 2023). As with AhR, several studies show certain metabolites of the KP are implicated in both depression and IBD, which is summarized in Table 3 (Paydas Hataysal et al., 2024). Given these connections, the relationship between AhR and KP could very well provide important insights into the comorbidity between IBD and depression. To better understand these links, this review will present the key metabolites of the KP and how they are implicated in IBD and/or depression (Table 4), with emphasis on known connections to AhR.

**Table 4:** List of KP metabolites and enzymes with known connections to IBD and/or depression.

Metabolite	KP enzyme	Implications in IBD	Implications in depression
N-formylkynurenine	*Tryptophan 2,3-dioxygenase (TDO) or Indoleamine 2,3-dioxygenase (IDO1/2)*	Unknown	Unknown
Kynurenine (KYN)	*Kynurenine Formamidase*	Aggravate (Michaudel et al., 2023; Zhao et al., 2022; Yu et al., 2024; Wnorowski et al., 2021)	Aggravate (Zhou M. et al., 2023; Deng et al., 2021)
Kynurenic acid (KYNA)	*Kynurenine aminotransferase (KAT) I-IV*	Ameliorate (Michaudel et al., 2023; Zhao et al., 2022; Radhakrishnan et al., 2025)	Ameliorate (Wang et al., 2024; Li Z. et al., 2022; Erabi et al., 2020; Zheng et al., 2022; Cheng et al., 2023; Lin et al., 2025; Ye et al., 2025; Colle et al., 2025) Aggravate (Brown et al., 2022)
3-hydroxykynurenine (3-HK)	*Kynurenine 3-monooxygenase (KMO)*	Unknown	Aggravate (Zhou M. et al., 2023; Lin et al., 2025; Ye et al., 2025; Yang et al., 2025; Yu et al., 2024)
Xanthurenic acid (XA)	*Kynurenine aminotransferase (KAT) II*	Ameliorate (Michaudel et al., 2023; Radhakrishnan et al., 2025)	Ameliorate (Bartoli et al., 2021; Farup et al., 2023; Molina-Carballo et al., 2021)
Anthranilic acid (AA)	*Kynureninase (KYNU)*	Ameliorate (Zhou F. et al., 2023)	Inconclusive (Murata et al., 2025; Pawlowski et al., 2021; Steiner et al., 2021)
3-hydroxyanthranilic acid (3-HAA)	*Kynureninase (KYNU)*	Inconclusive (Radhakrishnan et al., 2025; Huhn et al., 2020)	Aggravate (Deng et al., 2021; Xu et al., 2024)
Cinnabarinic acid (CA)	*Non-enzymatic conversion*	Unknown	Unknown
2-amino 3-carboxymuconic semialdehyde (ACMS)	*3-Hydroxyanthranilate-3,4-dioxygenase (3-HAO)*	Unknown	Unknown
2-aminomuconic 6-semialdehyde	*2-amino-3-carboxymuconic-6-semialdehyde decarboxylase*	Unknown	Unknown
Picolinic acid (PA)	*Non-enzymatic conversion*	Ameliorate (Radhakrishnan et al., 2025)	Ameliorate (Deng et al., 2021; Paul et al., 2022; Trepci et al., 2021; Sha et al., 2021; Yilmaz et al., 2022; Colle et al., 2025)
Quinolinic acid (QA)	*Spontaneous conversion*	Aggravate (Michaudel et al., 2023; Mohammadgholi-Beiki et al., 2024; Radhakrishnan et al., 2025)	Aggravate (Zhou M. et al., 2023; Deng et al., 2021; Kumar Arora et al., 2022; Bansal et al., 2022)
Nicotinic acid ribonucleotide	*Quinolinate phosphoribosyltransferase (QPRT)*	Unknown	Unknown
Nicotinic acid adenine dinucleotide	*Non-enzymatic conversion*	Unknown	Unknown
Nicotinamide adenine dinucleotide (NAD+)	*Non-enzymatic conversion*	Ameliorate (Kim et al., 2025; Novak et al., 2023; Hong et al., 2022) Aggravate (Gerner et al., 2018)	Ameliorate (Xie et al., 2025; Md et al., 2024; Barrong et al., 2024; Chen et al., 2024)

## 5 Kynurenine pathway (KP)

The KP is a metabolic pathway that is responsible for the breakdown of 95% of dietary Trp with the remainder largely used for the synthesis of serotonin (Boadle-Biber, 1993) and is summarized in Figure 2. This balance of Trp acquisition helps to support KYN-dependent serotonin deficiency hypothesized to play a role in MDD (Lapin and Oxenkrug, 1969), and the basis of the KYN hypothesis of depression (Lapin, 1973). The KP is activated by peripheral immune response or inflammation and has been associated with depression in adults (Haroon et al., 2020; Marx et al., 2021) and adolescents (Ozturk et al., 2021). Although, results from separate studies are inconclusive (Milaneschi et al., 2021). Modulation of the components (e.g., enzymes) involved in production of these metabolites, or even metabolite imbalances itself, are actively being discussed in the context of depression research as reviewed by Ou et al. (2023).

**Figure 2:**
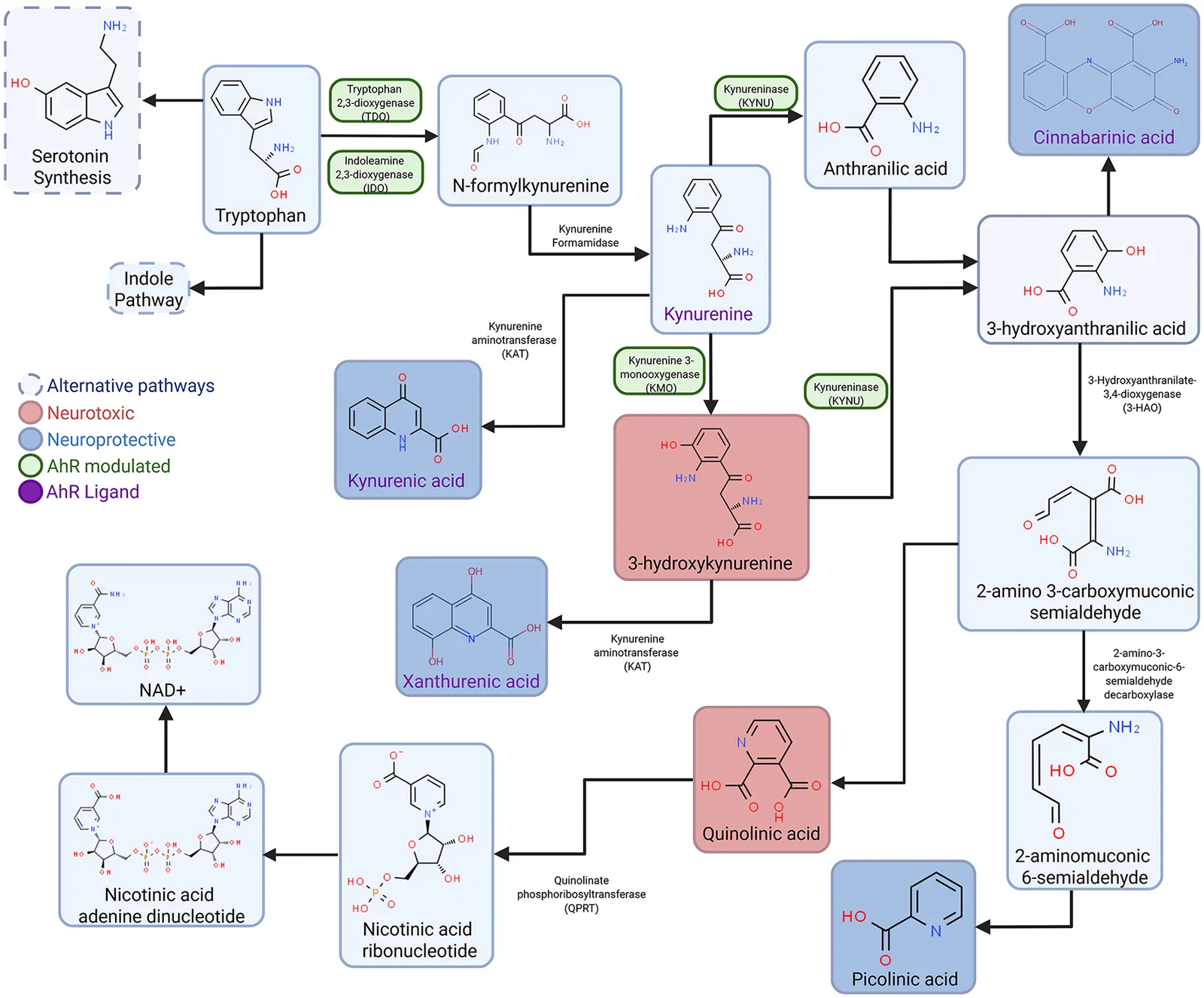
Summary of the kynurenine pathway and key components linked to the aryl hydrocarbon receptor. Tryptophan (Trp) is an essential amino acid that can be catabolized via several pathways such as serotonin synthesis, the indole pathways, or the kynurenic pathway (KP). Nearly 95% of dietary Trp follows the KP. Several metabolites of the KP are neuroactive such as the neuroprotective kynurenic acid (KYNA), xanthurenic acid (XA), and picolinic acid (PA), or the neurotoxic 3-hydroxykynurenine (3-HK), and quinolinic acid (QA). The aryl hydrocarbon receptor (AhR) is intimately entwined within the KP, inducing expression of tryptophan 2,3-dioxygenase (TDO) in the liver and indoleamine 2,3-dioxygenase (IDO) extrahepatically. In addition, both kynurenine 3-monooxygenase (KMO) and kynureninase (KYNU) are modulated by AhR, implying that AhR activation preferentially accelerates tryptophan degradation along the neurotoxic arm of the KP to provide additional reserves of the bioactive metabolite nicotinamide adenine dinucleotide (NAD+). There are also numerous KP metabolites which are ligands of the AhR such as: kynurenine (KYN), KYNA, XA, and cinnabarinic acid (CA). Created with Biorender.com.

### 5.1 Kynurenine (KYN)

Trp is an essential amino acid derived from dietary intake and can be utilized as a precursor for indole metabolism (Su et al., 2022), serotonin synthesis (O'Mahony et al., 2015), or the KP (Roth et al., 2021). Dietary Trp is converted by indoleamine 2,3-dioxygeenase (IDO) in the gut or tryptophan 2,3-dioxygenase (TDO) in the liver to eventually create KYN. KYN acts as the central point for the KP where several major bioactive molecules are synthesized from it. High concentrations of KYN can promote a pro-inflammatory state in the brain resulting in oxidative stress and further metabolism of neurotoxic metabolites as part of the KP (Wurfel et al., 2017). KYN is also a known AhR ligand and can pass through the BBB under homeostatic conditions with assistance from neutral amino acid transporters (Gal and Sherman, 1980). AhR activation with TCDD has been shown to induce expression of IDO1 and IDO2 in immune cells, implying downstream KP metabolites could potentially be modulated through the AhR (Vogel et al., 2008). Increases in IDO expression is known to assist in gut mucus barrier integrity (Alvarado et al., 2019). In fact, the KYN-AhR axis is hypothesized to play a role in IBD (Ala, 2022). Interestingly, exogenous KYN administration was shown to induce depressive-like behavior independently of IDO and large-scale immune activation (O'Connor et al., 2009). Lin S. et al. (2022) found that AhR expression was inhibited in a preclinical stress induced depressive-like behavior model while IDO1 was increased, resulting in elevated serum KYN levels. Activation of the AhR using crocetin reduced IDO1 expression, KYN levels, attenuated depression-like behavior, and potentially created additional endogenous AhR ligands via engagement of the indole metabolic pathway as shown by an increase of tryptophanase. As such, KYN and its association with AhR are promising relationships to explore in the context of comorbid IBD and depression.

### 5.2 Kynurenic acid (KYNA)

KYNA is derived from KYN via kynurenine aminotransferase (KAT) which is primarily expressed by astrocytes in the brain, select cells in the periphery, and certain bacterial species in the gut (Guillemin et al., 2001). A known AhR ligand, KYNA was shown to stimulate g protein coupled receptor (GPR)-35 expression on immune cells to induce anti-inflammatory (Fallarini et al., 2010). KYNA is associated with brain regions related to emotion and reward processing, but imbalances appear to contribute to mental health illnesses like schizophrenia (Erhardt et al., 2003), MDD (Ogyu et al., 2018), and BPD (Marx et al., 2021). To this point, a metabolomic analysis showed that KYNA was lower in human patients with MDD but correlated with improved responses to escitalopram therapy (Erabi et al., 2020). Interestingly, KYNA is unable to readily cross the BBB (Varga et al., 2016). However, increased production of KYNA reduces the availability of circulating KYN, which could potentially limit the neuro-inflammatory influence of KYN trafficking across the BBB (Pedersen, 2019). In preclinical animal models, low-dose levels of KYNA were used therapeutically to facilitate learning, memory (Martos et al., 2022), as well as ameliorate depressive-like behavior (Tanaka et al., 2020a). While not as extensive as mental health studies, results did show that healthy human control patients had a larger KYNA/KYN ratio when compared to UC and CD patients (Paydas Hataysal et al., 2024). It is important to note that KYNA has also been shown to have detrimental effects, with elevated KYNA production leading to CD4+ T cell exhaustion for example (Rad Pour et al., 2019). Currently, there remains no consensus in the literature with respect to the protective or deleterious influences of KYNA, and therefore further studies need to be conducted to determine how modulation of KYNA, possibly through AhR, impacts IBD and/or depression.

### 5.3 Quinolinic acid (QA)

Kynurenine 3-monooxygenase (KMO) acts as the rate limiting enzyme for the production of QA in the KP (Crozier and Moran, 2007). Brain-resident microglia and adipose tissue resident macrophages express high levels of KMO and can produce QA without stimulation (Favennec et al., 2015), but is dramatically increased in the presence of inflammatory mediators (Tanaka et al., 2020b). QA can be degraded by quinolinate phosphoribosyltransferase (QPRT) and eventually converted into nicotinamide adenine dinucleotide (NAD+), a critical component for several biochemical pathways in the body. With respect to the gut microbiome, certain microbes can potentially create QA independent of mammalian host cells such as *Bacteroidetes, Actinobacteria*, and *Proteobacteria* (Kaur et al., 2019). A recent fecal microbiome study determined that while over 90% of gut microbial genomes had the potential to degrade QA, less than 5% contained genes related to KYN degradation (Valles-Colomer et al., 2019). Concentrations of QA in the brain are typically very low in homeostatic conditions but can quickly increase to cytotoxic levels linked to a multitude of deleterious effects (Guillemin, 2012; Lugo-Huitron et al., 2013) and neuroinflammation (Lucido et al., 2021; Yan et al., 2023).

Psychotherapy approaches have been shown to decrease QA levels in patients and improve depression symptoms, consistent with pharmaceutical interventions (Savitz et al., 2022). A recent study explored inhibition of KMO in an animal model of depression and observed a significant increase in BDNF, glial fibrillary acidic protein (GFAP), nuclear factor erythroid 2-related factor 2 (Nrf2), and amelioration of depressive-like behavior (Bansal et al., 2022). Elevated QA levels in the prefrontal cortex have been associated with decreased performance in working memory and visuospatial processing in systemic lupus erythematosus (SLE) (Anderson et al., 2021), a disease with a well characterized comorbidity with MDD (Kosa et al., 2022). Increased QA levels were found in the cerebral spinal fluid (CSF) of suicide attempters which correlated with elevated levels of IL-6 and increased suicidal intent (Erhardt et al., 2013), and in autopsy studies of suicide victims (Steiner et al., 2011). Elevated QA levels have also been found in the CSF of traumatic brain injury (TBI) victims and were positively correlated with increased mortality (Meier and Savitz, 2022). One study found that American football players presented with increased QA levels following a concussion. In that study, researchers hypothesized repetitive concussions resulted in the exacerbation of QA production and associated depressive symptoms (Meier et al., 2021). In fact, QA detection has shown promise as a potential biomarker for ketamine efficacy and symptom amelioration for severe treatment resistant depression (Verdonk et al., 2019).

While the relationship between QA and depression is more established, there is also evidence that QA could potentially be involved in the pathogenesis of IBD. In a recent randomized control trial (RCT) study with diet induced pediatric CD remission patients, a reduction in QA was associated with amelioration of disease symptoms (Ghiboub et al., 2023). Shi et al. (2024) also reported that inhibition of KMO alleviated intestinal inflammation and suppressed pro-inflammatory IL-1β signaling in epithelial cells in a preclinical IBD DSS-induced colitis murine model. In fact, genetic KMO KO mice were found to be protected against TNBS-induced colitis in mice (Tashita et al., 2020). Collectively these studies illustrate that high levels of QA are associated with depression and IBD symptoms in clinical humans and preclinical animal models. Reducing QA, either by inhibition of KMO or substrate scarcity, could potentially protect against both conditions, and provide novel therapeutic targets for treatment. However, unlike KP metabolites such as KYNA, regulation of QA directly by AhR has not been established.

## 6 The microbiota gut brain axis (MGBA)—mechanisms connecting IBD and depression and implicated roles of AhR and/or the KP pathway

Despite growing evidence that IBD and depression are connected, the question remains as to what underlying mechanisms contribute to their comorbidity, and whether AhR and the KP contribute to this. Recent reviews on the comorbidity of IBD and depression highlight key mechanistic features which link these two disease states, which include microbial dysbiosis/metabolic imbalance, dysregulated immune responses/intestinal barrier disruption, systemic inflammation, and lastly BBB disruption and neuroinflammation (Simoes et al., 2026; Camara et al., 2026). For the purposes of this review, these key mechanistic MGBA components linking IBD and depression will be briefly described. In addition, where applicable, known roles of AhR and/or the KP in the regulation of these mechanisms linked to comorbid IBD and depression will be noted.

### 6.1 Microbial dysbiosis and metabolic imbalance

It is well known that microbial dysbiosis, a term to describe deleterious alterations in the residing microbiota and the metabolites, contributes to the pathogenesis of IBD (Haneishi et al., 2023). Notably, fecal analysis has consistently shown a reduction in bacterial diversity coinciding with an increase of pathogenic species in human IBD patients and preclinical animal models of IBD (Chen et al., 2014; Morgan et al., 2012). In addition, a cross-sectional study by Hu et al. (2023) found that decreased gut microbial diversity was closely related to MDD severity, and another integrative study found that the interactions between the gut microbiome and IBD had a significant effect on increasing the risk of depression (Qin et al., 2022). Patients diagnosed with either depression or IBD have consistently demonstrated distinct microbiomes as compared to healthy controls (Amos et al., 2021; Zhu et al., 2022; Li X. et al., 2022; Valles-Colomer et al., 2019). There is experimental evidence that alterations in the gut microbiome, or its absence, impacts behavior (Pouranayatihosseinabad et al., 2023). For example, the absence of a gut microbiome in germ-free (GF) mice displayed reduced anxiety-like behavior and increased motor activity which was restored following fecal microbiota transplant (FMT) from specific pathogen free (SPF) mice (Diaz Heijtz et al., 2011). It has also been shown that FMT from stressed mice to stress naïve mice (Li et al., 2019), and from human MDD patients to rats (Knudsen et al., 2021), could result in depressive- and anxiety-like behavior in recipient rodents. Recently, a case study by Doll et al. (2022) used FMT as an adjunctive therapy for two patients suffering from MDD and both patients reported symptom improvement 4 weeks after transplantation. Another such study found that 50% of MDD patients experienced GI disturbances, and enema-delivered FMT to be a tolerable and safe treatment strategy (Green et al., 2023). Collectively, these studies provide evidence that the gut microbiome plays a significant role in the relationship between IBD and depression, and alterations in a “healthy microbiota” can lead to metabolite destabilization.

During microbial dysbiosis, several metabolites involved in intestinal and brain health can be altered, which include short chain fatty acids (SCFAs), bile acids (BAs), and microbially derived vitamins (Simoes et al., 2026). Several studies have found that patients with IBD, depression, and active IBD with depression have reductions in butyrate producing bacteria (Franzosa et al., 2019; Thomann et al., 2022; La Rosa et al., 2019; Radjabzadeh et al., 2022), whereas the presence of butyrate producing bacteria, like *Faecalibacterium* and *Coprococcus*, have been associated with higher QoL in human patients with depression (Valles-Colomer et al., 2019). Butyrate, a SCFA produced from fermentation of dietary fibers, is crucial in the gut for enterocyte division, but, in the brain, butyrate has been shown to increase levels of brain-derived neurotrophic factor (BDNF), and low BDNF levels are linked to impaired neural plasticity often associated with depression (Cavaliere et al., 2022). Butyrate is primarily generated from species of the Firmicutes phylum, such as the *Roseburia* species, and has been shown to be consistently downregulated in IBD (Machiels et al., 2014; Franzosa et al., 2019; La Rosa et al., 2019; Recharla et al., 2023). A study by Zhu et al. (2018) showed that administration of *Roseburia intestinalis* to TNBS-colitis mice displayed an inhibition of IL-17 excretion and promoted T-reg differentiation which reduced IBD severity. In addition to SCFAs, disruption in BA synthesis, particularly secondary BAs, have been linked to IBD-related mood disorders (e.g., depression) and neuroinflammation (Zhao et al., 2025), detailed later in this review.

AhR is well-known to impact the microbiome, either through alterations in the microbiota community profile or the resulting metabolome (Yang et al., 2020). Currently, research has shown that AhR can regulate the microbiome through immune cell regulation and cellular signaling (e.g., IL-22), which includes the production of anti-microbial peptides, such as α/β-defensins and regenerating family member gamma (RegIII) members (Ghosh et al., 2026; Palrasu et al., 2025a,b; Fukumoto et al., 2014). For specific metabolites, AhR has been shown to enhance SCFA such as butyrate and vitamin D3 (Busbee et al., 2020; Huang and Huang, 2023), as well as alter BA production in the liver (Han et al., 2023). In addition, alterations in the microbial profile and resulting metabolome can impact AhR itself. For example, butyrate also serves as an endogenously produced ligand for AhR resulting in the transcription of AhR-dependent genes in intestinal epithelial cells (Marinelli et al., 2019). Indeed, many products of the gut metabolome provide ligands for the AhR such as bacterial metabolism of the essential amino acid Trp (Neavin et al., 2018). Of interest, one such study found that mice deficient in caspase recruitment domain 9 (Card9), an IBD susceptibility gene, presented with microbial dysbiosis and failure to catalyze Trp into AhR ligands. This resulted in a reduction of IL-22 and increased susceptibility to DSS-colitis (Lamas et al., 2016). Trp is broken down by gut microbes via the indole pathway, producing AhR ligands such as IAA and IPA (Dong and Perdew, 2020), and by mammalian cells mainly through the KP. Clinical studies show dysregulation of Trp metabolism can alter downstream metabolites of the KP, which are associated with IBD (Nikolaus et al., 2017), depression (Lapin, 1973), and comorbid IBD-depression (Lai et al., 2023).

### 6.2 Dysregulated immune responses and intestinal barrier disruption

It remains unclear if microbial dysbiosis and metabolic destabilization alter immune responses, or vice versa, during intestinal inflammation. However, enhanced immune cell activation leading to increased permeability or intestinal barrier disruption is a hallmark feature of IBD. Several components of a dysregulated immune system have been implicated in the pathogenesis of IBD, particularly the various subsets of immune cells involved in innate and adaptive responses. Recently, intestinal M1 and M2 macrophages were found to play a role in UC and CD pathogenesis. Macrophages isolated from IBD patients were shown to highly express M1 genes and inflammatory pathways, but only CD isolated macrophages were also associated with M2 genes (Dharmasiri et al., 2021). In some cases, such as in UC patients, neutrophil migration contributes to the exacerbation of this disease (Bressenot et al., 2015), likely through localized production of ROS and pro-inflammatory cytokines like IL-8 (Mouzaoui et al., 2014). In CD, there is evidence that supports a neutrophil function and recruitment as a contributing factor (Therrien et al., 2019). Since neutrophils play a critical role in anti-microbial surveillance and defense, their role remains crucial in maintaining gut homeostasis. A recent study identified neutrophils had the ability to “self-correct” their responses to bacteria and prevent excessive aggregation to maintain organ homeostasis (Kienle et al., 2021). ILC3s play a critical role in maintaining intestinal homeostasis via increased production of IL-22 (Longman et al., 2014). A study on immune cell populations in human IBD patients showed ILC3 populations were reduced in UC mucosa compared to CD, where this population was enriched (Mitsialis et al., 2020). DCs, classic examples of antigen presenting cells (APCs), were shown to accumulate in the lamina propria of IBD patients and impair lymphoid trafficking to follicles. This subsequently prevents antigen detection and regulation of the immune response and could contribute to IBD (Hart et al., 2005). DCs from IBD patients produce higher levels of the pro-inflammatory cytokines IL-6 and IL-12 which could influence downstream Th1 immune responses, and it has been shown that tolerogenic CD103+ CD11b+ are reduced in the inflamed tissue of CD patients (Faleiro et al., 2019). Furthermore, APCs mediate the differentiation of naïve T-cells to effector Th cells as a part of adaptive immune response. This differentiation helps maintain gut homeostasis and dysfunction of this system is known to contribute to IBD development (Wallace et al., 2014). Historically, CD is suspected to be driven by Th1 immune responses whereas UC is thought to be driven by Th2 responses (Lee et al., 2025; Krzystek-Korpacka et al., 2026). However, other findings suggest that Th17 plays a significant role in IBD generally (Lee et al., 2020). Th17 differentiation from naïve CD4+ T cells requires IL6, IL-1β, and transforming growth factor (TGF)-β, and this cell type is known to produce the cytokines IL-17, IL-21, IL-22, interferon (IFN)-γ, and TNF-α. The so called “IL23-IL17 Immune Axis” has been identified as a mechanistic contributor in both UC and CD (Noviello et al., 2021; Schmitt et al., 2021). Other studies have highlighted Th17 produced cytokine IL-22, which is also produced by ILC3s, plays an important role in the attenuation of IBD symptoms, often described as a “double agent” as its pro- or anti-inflammatory actions depend on the local cytokine environment (Mizoguchi et al., 2018). While the exact immune cell response dysregulation might differ among IBD patients, due to the enhanced inflammation often seen in IBD patients, intestinal barrier disruption is often the result, which leads to complications such as reduced protective luminal mucus production and increased intestinal permeability (Dunleavy et al., 2025).

Depression has also been linked to dysregulated immune responses (e.g., inflammation) and disruption in the intestinal barrier (Trzeciak and Herbet, 2021; Kohler et al., 2017). The hypothalamic-pituitary-adrenal axis (HPA) and components of the efferent nervous system (ENS) and afferent nervous system (ANS), such as the vagus nerve (VN), are all known to interact with the immune system (Foster et al., 2017). However, it is important to note that inflammation in depression, particularly in the absence of an inflammatory comorbid condition such as IBD, remains debatable (Luo et al., 2025).

In several studies highlighted in Table 2, treatment with AhR ligands often results an anti-inflammatory immune response (e.g., increased Tregs vs. Th17/Th1) and enhanced intestinal barrier function (e.g., increasing expression of tight junction proteins). For example, resveratrol has been shown to have anti-inflammatory properties and alleviated intestinal barrier dysfunction through AhR activation in preclinical murine IBD models (Pan et al., 2020; Singh et al., 2010). This was also reflected in a porcine model challenged with diquat. Weaned piglets were administered increasing concentrations of diquat (i.p) to induce intestinal inflammation and damage. In this study, resveratrol maintained intestinal integrity, prevented oxidative stress, and reduced intestinal inflammation through activation of AhR (Xun et al., 2021). In addition, pretreatment with two doses of TCDD (15 μg/kg) led to a reduction in weight loss, colonic inflammation, and an increase of Tregs in an AhR-dependent manner using a preclinical 2,4,6-trinitrobenzenesulfonic acid (TNBS)-induced IBD model (Benson and Shepherd, 2011). In line with this finding, a single dose of TCDD (25 μg/kg) was shown to attenuate clinical symptoms of IBD in mice administered 3% DSS and treated AhR KO mice were shown to have dysregulation in Treg differentiation (Singh et al., 2011).

### 6.3 Systemic inflammation

Systemic inflammation, marked by increases in pro-inflammatory cytokines and bacterial products (e.g., LPS) in the blood circulation, are well known extraintestinal complications in IBD (Caballero-Mateos et al., 2025; Di Vincenzo et al., 2024). While debatable, as noted in the previous section, systemic inflammation has also been linked to depression, often associated with elevated biomarkers such as C-reactive protein (CRP), interleukins (IL-6, IL-1β), tumor necrosis factor-alpha (TNF-α), and certain immunoglobulins (Perry et al., 2021; Dowlati et al., 2010; Cheng et al., 2026; Zhou et al., 2025). In fact, neutralizing IL-6 antibodies were found to improve symptoms of depressed mood in patients with rheumatoid arthritis (RA) (Sun et al., 2017). Ibrutinib, a bruton's tyrosine kinase inhibitor, was administered in a preclinical murine depression model and resulted in reduced neuroinflammatory markers and increased the expression of ROS protective enzymes, leading to amelioration of depression-like symptoms (Li et al., 2021). MDD and BPD patients have increased risk of developing multiple sclerosis (MS), an inflammatory disorder of the CNS, and MS patients also have an increased risk of developing these psychiatric illnesses (Ozsimsek et al., 2026). Perinatal or post-partum depression has been the subject of study for decades and inflammation has been implicated as major contributing factor in this condition. A recent study showed that women 2–3 years post-delivery who had clinically relevant depression, anxiety, and/or post-traumatic stress disorder (PTSD) had higher levels of genes associated with inflammation (Nicoloro-SantaBarbara et al., 2022). However, while other studies are in support of increased inflammation in post-partum patients (Xie, 2026), others show conflicting or non-correlative results (Cohen et al., 2026; Kaplan et al., 2026).

While a majority of preclinical IBD models studies using AhR ligands often focus on impacts on localized intestinal inflammation, there is evidence that circulating inflammatory biomarkers are reduced, such as serum amyloid A (SAA) (Wei et al., 2025; Busbee et al., 2020). This could be an indirect consequence of regulating localized gut inflammation and intestinal barrier homeostasis. However, in the CUMS model of depression, treatment with I3C was able to suppress circulating levels of pro-inflammatory cytokines presumably in the absence of gut inflammation (Pan et al., 2022). In any case, if AhR is shown to reduce localized intestinal inflammation, then a reduction in systemic inflammation driven by the former can still be an indirect beneficial outcome that is capable of reducing extraintestinal negative outcomes, including mood disorder development such as depression.

### 6.4 BBB disruption and neuroinflammation

The BBB lies between the cerebral capillary blood and the interstitial fluid of the brain primarily composed of capillary endothelial cells, the basement and neuroglial membrane, and glial podocytes (Daneman and Prat, 2015). This barrier simultaneously protects the brain from invasion of pathogens, toxins, and host-immune cells while regulating the entry of necessary metabolites. This task is accomplished by highly specific embedded transport proteins in the basement membrane and cellular enzymes which function to alter substances for passage through the cells (Kadry et al., 2020), which allows only select substances to pass the BBB. Pathological conditions, like chronic inflammation, have been shown to compromise the barrier and allow passage of molecules that would normally not be permitted (Haruwaka et al., 2019). Preclinical animal models of IBD have shown increased intestinal and BBB permeability resulting in neuroinflammation, defined by activation of neuronal cells such as microglia and astrocytes, and subsequent brain dysfunction (Mitchell et al., 2022; Kelly et al., 2016; Hathaway et al., 1999; Han et al., 2018; Morton et al., 2026). Furthermore, mice challenged with DSS and treated with niacin showed amelioration of IBD and depression-like symptoms in part due to increased hippocampal levels of tight junction proteins zonula occluden- (ZO)-1, occludin, and claudin-5 (Wadie et al., 2023). Findings from Wu et al. (2023) also showed that *Bacteroides vulgatus* and its key metabolite p-hydroxyphenylacetic acid (4-HPAA) alleviated DSS-induced colitis and depression-like behavior in preclinical murine models, potentially by increasing the expression of claudin-5 in the vascular endothelium of the BBB. In another preclinical study, DSS administered mice showed a reduction in claudin-5 and occludin in colonic tissue which correlated with increased neuroinflammation characterized by microglia activation in the prefrontal cortex (Silveira et al., 2023). Beyond direct gut inflammation, animal studies also suggest that chronic social stress alters BBB integrity, promotes depression, and increases intestinal permeability (Menard et al., 2017; Hao et al., 2024; Schneider et al., 2023). A recent publication from Peng et al. (2022) found that in a preclinical chronic stress-induced depression model, disruption in the BBB led to the recruitment of peripherally-derived IL-17 producing Th-17 cells, and ultimately resulted in neuroinflammation.

There is evidence that AhR is expressed in barrier organs/cells such as the brain endothelial cells (You et al., 2019), with one study finding that administration of TCDD increased ATP-driven xenobiotic efflux transport at the BBB (Wang et al., 2011). In addition, AhR ligand ILA was found to reduce neuroinflammation in the CUMS model of depression as highlighted in Table 3. Reduction in neuroinflammation in this study was defined in the Qian et al. (2024) report to be reductions in microglia activation and pro-inflammatory cytokines in the brain. As noted, activation of microglia is a hallmark of neuroinflammation and this often results in the imbalance of KP metabolites, particularly in increased production of neurotoxic QA (Yan et al., 2023). Interestingly, in the brain of aged rats, AhR expression is decreased, which was correlated with increased microglia activation (Sun et al., 2026).

## 7 Summary and future considerations

The relationship between IBD and depression is an ongoing field of active research, and the mechanisms related to their comorbidity and potential treatments remain underdeveloped. This review attempts to provide a summary of the growing evidence that AhR and the KP could play critical roles in better understanding this disease comorbidity and develop new therapeutic strategies. Based on the current highlighted literature, there is certainly an argument to be made that metabolites of the KP hold significant influence in the emergence of depression in the IBD population, and that the modulation or regulation of AhR could be exploited as a potential therapeutic target.

Highlighted in this review are key mechanisms linked to IBD, depression, and IBD-related depression shown to be impacted either directly or indirectly through AhR, including microbial dysbiosis/metabolic imbalance, dysregulated immune response/intestinal barrier disruption, systemic inflammation, and BBB disruption/neuroinflammation. Most importantly, the bidirectional link between the disorders appears to be an imbalance of Trp metabolism that promotes neurotoxic KP metabolite production, such as QA. As noted elsewhere in this review, it is well established that during depression or depressive-like behavior in preclinical models there is a shift from neuroprotective KYNA to neurotoxic QA in the CNS. While Nikolaus et al. (2017) established that IBD patients had evaluated QA in their serum compared to healthy controls, what was not known until recently was whether this KP shift was present during IBD particularly within the GI tract. This is an important distinction as Yuan et al. (2021) established that IBD patients with depression/anxiety (e.g., UC) had a significantly altered serum metabolic profiles when compared to healthy controls and IBD patients without depression/anxiety. However, recently published metabolomics and transcriptomics results in advanced IBD patients from Wehkamp et al. (2026) show that mucosal and serum samples are dominated by increased QA production that results in decreased NAD+ synthesis. As noted by the researchers in this study, IBD patients had advanced Trp degradation that led to the accumulation of QA which was combined with reduced QPRT expression. Reduced QPRT, the enzyme that metabolizes QA, results in sustained inflammation due to the decrease in NAD+ synthesis. Wehkamp et al. provided proof of this by showing that QPRT knockdown resulted in sustained inflammation, while NAD+ precursor supplementation resulted in reduced inflammation in a preclinical IBD mouse model.

With this new data showing QA accumulation in the intestinal mucosa and serum, combined with other studies highlighted in this review, a working hypothesis involving the AhR/KP axis in comorbid IBD and depression can be reasonably postulated from a bidirectional standpoint. From IBD to depression, it is well known that IBD patients have intestinal inflammation and metabolic imbalance. What was recently discovered was a net result of increased intestinal QA accumulation that allows for sustained inflammation. This sustained inflammation leads to intestinal permeability that allows for the circulation of inflammatory mediators (cytokines, LPS) and neurotoxic metabolites (e.g., QA from Trp during KP). QA in circulation, combined with other inflammatory mediators, can promote the breakdown of the BBB, as in addition to being a known neurotoxin, QA is also a disruptor of the BBB (St'astny et al., 2000). As the BBB is disrupted, inflammatory mediators in combination with increased circulating QA promotes neuroinflammation and increased incidence of potential depression. Taken from the other direction of depression to IBD, if stress/depression results in neuroinflammation, CNS-derived QA can increase due to neuronal cell activation (e.g., microglia). If allowed to accumulate, QA will promote neurotoxic effects and disruption of the BBB, allowing for increased circulation of proinflammatory mediators and QA from the CNS to routes leading directly to the intestine or indirectly through gut-brain connections, such as simulation of the vagus nerve. Inasmuch, regulation of Trp metabolism, and more specifically KP is crucial in maintaining homeostasis. AhR is well known to both regulate and be regulated by either enzymes of the KP pathway or by KP metabolites, as noted in Figure 2. For example, during IBD and/or depression, a shift of Trp metabolism in the KP from decreased KYNA to QA will result in decreased activation of AhR (e.g., KYNA as AhR ligand), which has been shown to promote protective effects in both disorders. In addition, if AhR activation is increased either through dietary modulation or some form of therapeutic targeting, AhR can impact KP enzyme activity (e.g., IDO, KMO, KYNU) to shift away from enhanced production of QA or even alter NAD biosynthesis (Wang et al., 2013) to suppress inflammation. While a promising hypothesis based on the current research results, future key experiments will need to be conducted to establish QA dysregulation as a key mechanism linked to comorbid IBD and depression, in addition to determining if AhR can regulate either directly or indirectly this KP-QA shift.

Taken altogether, Figure 3 highlights key events that link IBD with depression in a bi-directional manner, with special emphasis on KP dysregulation within the GI tract and CNS. In addition, this summary figure highlights AhR protective effects noted in this review which are linked to key components of the MGBA, with noted lack of evidence for its role in direct KP-QA regulation. Lastly, there are some current limitations and future considerations that should be taken into account as it relates to the topic of AhR/KP in IBD and depression comorbidity, as discussed below.

**Figure 3:**
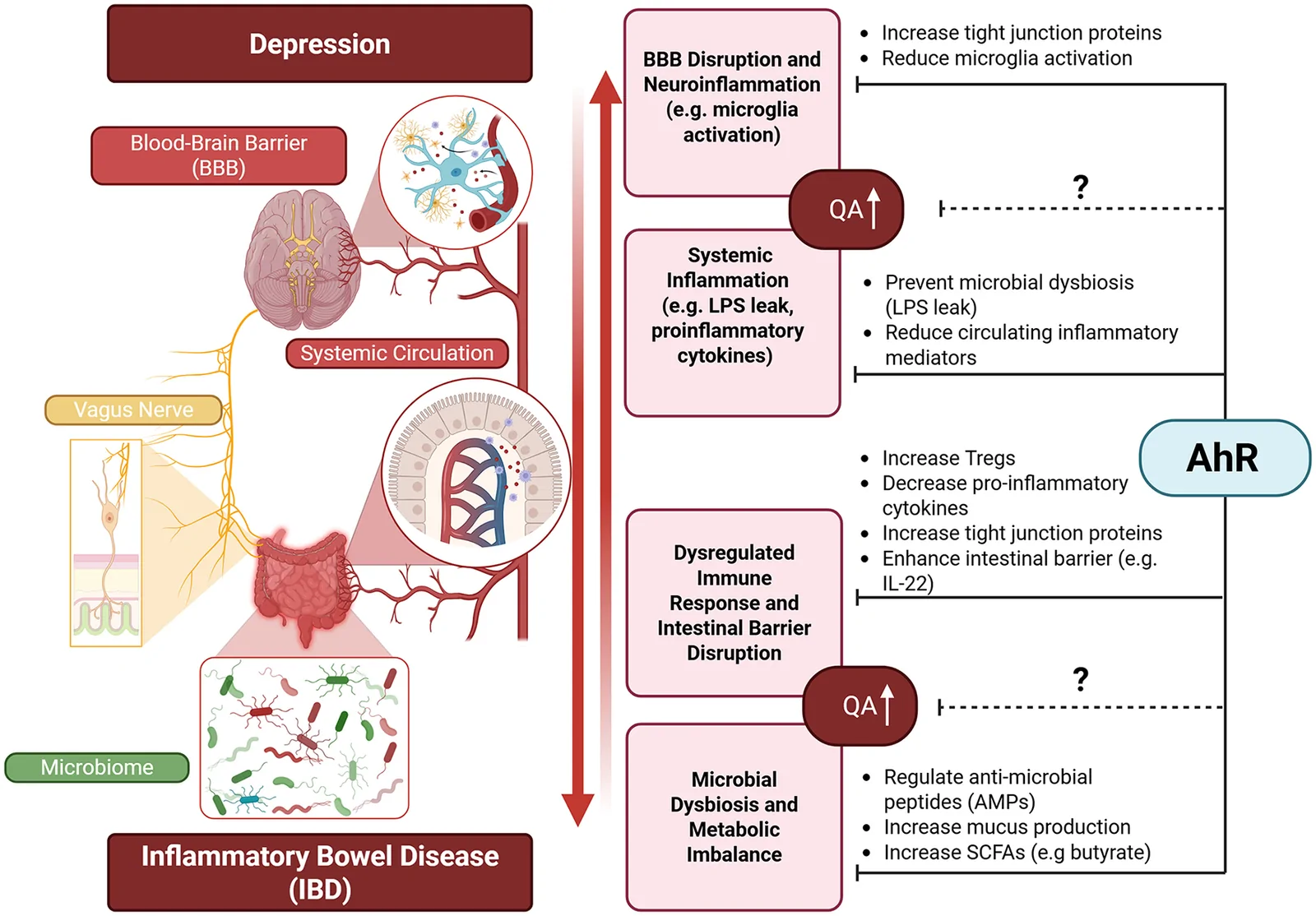
Key mechanisms of the microbiota-gut-brain-axis associated with comorbid IBD and depression and implications of regulation by AhR. The microbiota-gut-brain-axis (MGBA) is a bidirectional communication between the gut microbiome, vasculature, nervous system, and brain. IBD and IBD-related associations with depression involve key mechanism of the MGBA, which include: microbial dysbiosis/metabolic imbalance, dysregulated immune response/intestinal barrier disruption, systemic inflammation, and BBB disruption/neuroinflammation. In a disease state such as IBD, microbial dysbiosis and inflammatory responses can increase intestinal permeability (“leaky gut”). With increased intestinal permeability, systemic inflammation and circulation of destabilized can alter the BBB permeability (“leaky brain”). With the primary defensive measure mitigated, neuroactive molecules (e.g., QA), pro-inflammatory cytokines, and activated immune cells can infiltrate the once “immune privileged” CNS and promote neuroinflammation (e.g., activated microglia and further promote neurotoxic KP metabolites such as QA). AhR has been shown to regulate or mitigate many of these MGBA mechanisms linked to comorbid IBD and depression, although future studies will need to be conducted to determine if AhR directly or indirectly can regulate neurotoxic QA accumulation. Created with Biorender.com.

### 7.1 Human clinical data/trials and investigation of comorbid IBD/depression

While this review provides some human data as it relates to AhR/KP and IBD/depression, most of the studies highlighted are limited to preclinical animal models. As of the writing of this review, a current search of clinical trials (clinicaltrials.gov), using the Medical Subject Headings (MeSH) terms “inflammatory bowel disease” (condition), “depression” (other terms), and “aryl hydrocarbon receptor” (intervention/treatment) yield no applicable trials. In fact, despite a wealth of data showing the effectiveness of AhR ligands in preclinical IBD models, a MeSH search of “inflammatory bowel disease” (condition) and “aryl hydrocarbon receptor” (intervention/treatment), only one trial is noted (NCT03512327). NCT03512327 is classified as an interventional study to assess the efficacy of the autoimmune protocol (AIP) diet in IBD patients, with no direct or isolated effects attributed specifically to AhR. Similarly, despite evidence of the potential beneficial use of AhR ligands in preclinical models of depression, a MeSH search of “Depression Disorders” (condition) and “aryl hydrocarbon receptor” (intervention/treatment) yield no results. A search of human clinical trials for “inflammatory bowel disease” (condition) and “Depression Disorders” (other terms) does yield 288 results, so research in this area of comorbidity is active, just not in the context of AhR. It is important to note that there is currently one clinical trial (NCT01304355) related to “Depression Disorders” and “Kynurenine Pathway and Its Metabolites” in the context of irritable bowel syndrome (IBS), but this study is not related to AhR.

Reasons for the lack of human clinical data related to AhR/KP and comorbid IBD/depression could certainly be due to the challenges in drug development centered around AhR targetings, which will be discussed in the subsequent section. However, there is another limitation highlighted in the current review that should be taken into consideration, which is the lack of available studies that synergistically look at the impact of AhR/KP in the context of comorbid IBD and depression. As detailed in the current review, most studies will investigate the impact of AhR and KP in either the context of IBD or depression separately. It is certainly understandable given the complexity of both disease conditions, however, this makes it more difficult to determine what, if any, role these components play in either IBD alone, depression alone, or in comorbid IBD and depression. Inasmuch, future studies investigating the roles of AhR and KP would need to be more holistic and comprehensive, taking into account how regulation of these factors can potentially impact key mechanisms related to IBD and depression comorbidity.

### 7.2 Pharmacological interventions and drug development challenges

Despite numerous preclinical studies showing the efficacy of AhR ligands in a variety of inflammatory disorders, there is currently only one FDA-approved drug that is used specifically for the purpose of targeting AhR, which is Tapinarof (Bobonich et al., 2023). However, Tapinarof is a topical treatment only approved for use in plaque psoriasis and is not approved for use in either GI-related inflammation (e.g., IBD) or treatment of mood disorders (e.g., depression). There have been pharmacological strategies developed to target AhR for inflammatory GI conditions with some noted challenges, as reviewed by Faber et al. (2022). These approaches included the use of small molecules, dietary/microbial metabolites, AhR proteolysis targeting chimeras (PROTACs), and oligonucleotide-based approaches. Ran et al. (2026) also recently reviewed the potential of targeting AhR through natural product treatment in IBD. Both of these reviews acknowledge the promising results in preclinical animal models but also highlight difficulties in this strategy related to factors such a low bioavailability due to quick metabolism in the liver (e.g., effective doses in animal models exceed realistic concentrations from dietary sources), lack of efficacy and safety evidence in human-based studies (e.g., clinical trials), a wide-array of ligands of AhR with various affinities and contradicting outcomes in different tissue types/cells (anti-inflammatory vs. pro-inflammatory), and lastly, many AhR ligand studies lack evidence showing that the effects are mediated directly by AhR (e.g., transcriptional regulation). Taken together, future approaches will have to address these challenges and issues moving forward.

### 7.3 Biological sex as a variable

In addition to improved pharmacological interventions targeting AhR or KP in the context of comorbid IBD and depressions, biological sex should also be considered in future studies. Prevalence and pathogenesis of both IBD and depression have potential sex-dependent differences (Mohammadi et al., 2023; Xu et al., 2022). In addition, many studies note a potential interaction or crosstalk AhR has with estrogen receptors (ERs) (Matthews and Gustafsson, 2006), providing evidence AhR could have differential impacts related to biological sex. In fact, studies devoted to the sexual dimorphism of AhR have found several instances of altered expression and/or function, such as *in-utero* activation results in fetal alterations of gonadotropins, testicular steroidogenesis, and sexual behavior in adult males (Hattori et al., 2018). In addition, activation of AhR in males was shown to produce more female offspring in mice without altering paternal X/Y chromosome ratios (Bircsak et al., 2020). Transcriptomics analysis of hepatic genes also showed differential regulation between male and female C56BL/6 mice when exposed to AhR ligand TCDD (Lee et al., 2015). In another study of kidney disease, renal expression of AhR mRNA was 150% higher in Sprague-dawley female rats compared to males (Lu et al., 2006). As with AhR, regulation of the KP and its metabolites also were shown to have sex-specific outcomes related to depression (Nikkheslat et al., 2025). These noted sexual dimorphisms in AhR and KP are important in the context of health management of diseases such as IBD, depression, and comorbid IBD/depression, and should be considered in future studies.

### 7.4 Role of the vagus nerve (VN)

There are other important components of the MBGA related to comorbid IBD and depression that lack exploration of AhR and/or KP involvement, such as the VN. The VN has been shown to influence behaviors such as emotional bias (Johnson and Steenbergen, 2022), mood, effort, or motivation (Ferstl et al., 2024), all of which can be severely impacted in patients with MDD. The efferent fibers begin in the dorsal motor nucleus (DMN) and nucleus ambiguous, terminating at various organ systems allowing for widespread autonomic modulation (Pavlov and Tracey, 2017). Efferent VN fibers communicate with the ENS which reside in the submucosa and myenteric plexus of the gut (Dicks, 2022). Abrogation of vagal stimulation, like in the case of vagotomies, have shown promise in attenuation of depressive-like behavior (Yang et al., 2023), but it is worth noting that this also results in a positive correlation with a later diagnosis of IBD (Liu et al., 2020). However, there is a current lack of studies investigating how modulation of AhR and/or the KP could impact VN-mediated influences on comorbid IBD and depression, and as a potential key component of the MGBA, these current unknowns need to be addressed.
